# Can blood and semen presepsin levels in males predict pregnancy in couples undergoing intra-cytoplasmic sperm injection?

**DOI:** 10.12669/pjms.325.10610

**Published:** 2016

**Authors:** Ali Ovayolu, Cansev Yilmaz Arslanbuğa, Ismet Gun, Belgin Devranoglu, Arman Ozdemir, Sule Eren Cakar

**Affiliations:** 1 Dr. Ali Ovayolu, Department of Obstetrics and Gynecology, Cengiz Gokcek Public Hospital, Gaziantep, Turkey; 2 Dr. Cansev Yilmaz Arslanbuga, Department of Clinical Microbiology, Zeynep Kamil Education and Research Hospital, Istanbul, Turkey; 3 Dr. Ismet Gun, Associate Professor, Department of Obstetrics and Gynecology, GATA Faculty of Medicine, Istanbul, Turkey; 4 Dr. Belgin Devranoglu, Department of Obstetrics and Gynecology, Zeynep Kamil Education and Research Hospital, Istanbul, Turkey; 5 Dr. Arman Ozdemir, Department of Urology, Zeynep Kamil Education and Research Hospital, Istanbul, Turkey; 6 Dr. Sule Eren Cakar, Department of Clinical Microbiology, Zeynep Kamil Education and Research Hospital, Istanbul, Turkey

**Keywords:** ICSI, Infertility, Oligoasthenoteratozoospermia, Presepsin, Seminal plasma

## Abstract

**Objective:**

To determine whether semen and plasma presepsin values measured in men with normozoospermia and oligoasthenospermia undergoing invitro-fertilization would be helpful in predicting ongoing pregnancy and live birth.

**Methods:**

Group-I was defined as patients who had pregnancy after treatment and Group-II comprised those with no pregnancy. Semen and blood presepsin values were subsequently compared between the groups. Parametric comparisons were performed using Student’s t-test, and non-parametric comparisons were conducted using the Mann-Whitney *U* test.

**Results:**

There were 42 patients in Group-I and 72 in Group-II. In the context of successful pregnancy and live birth, semen presepsin values were statistically significantly higher in Group-I than in Group-II (p= 0.004 and p= 0.037, respectively). The most appropriate semen presepsin cut-off value for predicting both ongoing pregnancy and live birth was calculated as 199 pg/mL. Accordingly, their sensitivity was 64.5% to 59.3%, their specificity was 57.0% to 54.2%, and their positive predictive value was 37.0% to 29.6%, respectively; their negative predictive value was 80.4% in both instances.

**Conclusion:**

Semen presepsin values could be a new marker that may enable the prediction of successful pregnancy and/or live birth. Its negative predictive values are especially high.

## INTRODUCTION

Infertility is a frequently seen problem with a 10-15% rate of incidence, and is associated with male factors in as many as 50% of cases. A male infertility-associated factor is usually found together with abnormal semen parameters.^1^ One of these is idiopathic oligoasthenoteratozoospermia (iOAT). iOAT unattributable to any specific cause accounts for about 24% of cases. OAT is diagnosed in accordance with the World Health Organization and/or Kruger strict criteria.^2^,^3^ Presepsin is a newly discovered substance that can be measured in blood and semen, and has been found to increase in many infections and non-infectious immune events.^4^-^6^ Cluster of differentiation 14 (CD14) is a glycoprotein expressed on the membrane surface of monocytes and macrophages and serves as a receptor for lipopolysaccharides (LPSs) and LPS-binding proteins (LPBs).

During inflammation, plasma protease activity generates soluble CD14 (sCD14) fragments. One of them, the sCD14 subtype (sCD14-ST), or presepsin, is normally present in very low concentrations in the serum of healthy individuals and has been shown to increase in response to bacterial infections.^5^,^7^,^8^ Procalcitanin and inflammatory mediators such as IL8 have been investigated in male infertility.^9^ Additionally, in many sexually-transmitted infections, interleukins were studied in systemic and local genital secretions.^10^

Focusing on couples with unexplained infertility and iOAT, our aim in this study was to research whether presepsin values of semen and blood plasma samples provided by the male partner on the oocyte pick-up day (OPU) could predict biochemical pregnancy, successful pregnancy, and live birth. No male patient with an infectious illness on the sample day was included in the study.

## METHODS

This study was peformed in the Department of Obstetrics and Gynecology, Zeynep Kamil Training and Educational Hospital, between July 2013 and July 2014, among infertile couples that were diagnosed as having iOAT or unexplained infertility (normozoospermia) for which no female-attributed cause could be found. The women were aged between 23 and 39 years; the patients were non-smokers and had a body mass index (BMI) <28 kg/m^2^, and had no uterine, tubal or ovarian problems. Ovulation induction was performed using the classic long agonist protocol or fixed GnRH antagonist protocol. Both GnRH agonist and GnRH antagonist continued until the day of hCG administration to induce ovulation. On day 3 of cycle, gonadotropin treatment was initiated. The starting gonadotropin dose was individualized according to age, BMI, ovarian reserve determined by antral follicle count and basal FSH, and experience from previous cycles (between150-300 IU). Serial ultrasonographic checks and estradiol (E2) level measurements were peformed until 3 follicles were ≥17 mm and a serum E2 level>500 pg/mL were detected. Choriogonadotropinalpha 250 μg s.c. (Ovitrelle; MerckSerono, Turkey) was administered to induce final follicular maturation. Oocyte retrieval took place 35.5-36 h after hCG administration. Intra-cytoplasmic sperm injection (ICSI) was performed after four hours of OPU. Fertilization was assessed at 16-18 h after ICSI. All embryo transfers were performed after OPU in two to five days.

According to national policy, the best morphologic grade one or two embryos were transferred into the uterine cavity under ultrasound guidance (GE logic alfa 200). Luteal support was initiated on the night of oocyte retrieval and continued until the day of pregnancy testing. If the test was positive, progesterone treatment was continued up to the 9th gestational week. Biochemical pregnancy was defined as a positive pregnancy test result (hCG levels > 20 mIU/mL) 12 days after embryo transfer. Patients that could not undergo embryo transfer were excluded from the study.

Seminal plasma samples were given for ICSI on the OPU day after 3 or 4 days of abstinence, and venous blood samples were taken and centrifuged at 2000 rpm for 10 minutes, and stored at 80°C until the required day for analysis. After determining pregnancy test results and live birth outcomes, samples were dissolved in a few hours and final measurements were performed. Patients were divided into two groups according to pregnancy occurrence. Group-I comprised those who were pregnant and Group-II was for those without pregnancy. Presepsin was measured using a PATHFAST chemiluminesence immunoassay analyzer (Mitsubishi Medience, Tokyo, Japan). Presepsin values measured and compared between the blood and semen groups.

Statistical analyses were performed using the Statistical Package for the Social Sciences for Windows version 15.0 software (SPSS, Chicago, IL., USA). Descriptive statistics are given as mean, standard deviation, frequency, and percentage. A parametric comparison was performed using Student’s t-test, and a non-parametric comparison was conducted using the Mann-Whitney U test. Statistical significance was defined as p < 0.05.

### Ethics Committee Approval

Approval was received for this study from the ethics committee of Zeynep Kamil Education and Research Hospital and written informed consent was obtained from patients who participated in this study.

## RESULTS

In total, 114 patients were included in the study. There were 42 patients in Group-I and 72 patients in Group-II. Table-I indicates that there was no statistically significant difference between the groups in terms of demographic and treatment characteristics. The rate of ongoing pregnancy was 78.6%, and the rate of live birth was 66.7%. A comparative examination of the semen and plasma presepsin values revealed higher values in Group-I than in the non-pregnant Group-II in terms of biochemical pregnancy, successful pregnancy, and live birth. However, in terms of successful pregnancy and live birth, only semen presepsin values were statistically significantly higher in Group I than in Group II (p= 0.004 and p=0.037, respectively). Table-II shows that there is no statistical difference between the oligoastenospermic group and the normozoospermic group in terms of semen and plasma presepsin values.

**Table-I:** Demographic, stimulation, and treatment outcome characteristics, and seminal plasma and plasma presepsin values of the groups.

*Characteristics*	*Group I*	*Group II*	*P*
Womens’ age(years)	30.3±4.4	31.6±7.3	0.340*
Infertility duration, y	5.7±4.0	6.4±3.9	0.373*
FSH, mIU/ml	6.9±1.6	6.7±1.5	0.457*
Average used gonadotrophin, IU	2002.1±1001.8	2204.1±1033.8	0.325*
Mens’ age(years)	33.5±4.8	34.3±7.6	0.548*
Biochemical pregnancy, n	n (42)	n (72)	
Semen presepsin, pg/mL	541.6±837.6	270.5±364.3	0.060^b^
Plasma presepsin, pg/mL	343.2±217.3	358.6±206.1	0.458^b^
Ongoing pregnancy, n	n (33)	n (81)	
Semen presepsin, pg/mL	644.1±927.1	261.2±346.1	0.004^b^
Plasma presepsin, pg/mL	341.0±226.1	357.7±203.6	0.420^b^
Live birth, n	n (28)	n (86)	
Semen presepsin, pg/mL	643.0±973.2	280.0±367.7	0.037^b^
Plasma presepsin, pg/mL	343.2±225.4	356.2±205.1	0.521^b^

Data are presented as mean ± SD.

* Student’st-test and bMann-Whitney U test.

**Table-II:** Seminal plasma and plasma presepsin values in the oligoastenospermia and normozoospermia groups.

*Characteristics*	*Oligoastenospermia n (61)*	*Normozoospermia n (53)*	*P*
Mens’ age, years	33.3±5.3	34.9±8.0	0.182*
Seminal plasma Presepsin, pg/mL	371.6±490.4	366.3±695.75	0.829^b^
Plasma Presepsin, pg/mL	377.6±233.0	324.3±176.6	0.338^b^

Data are presented as mean ± SD.

* Student’st-test and bMann-Whitney U test.

**Fig.1:**
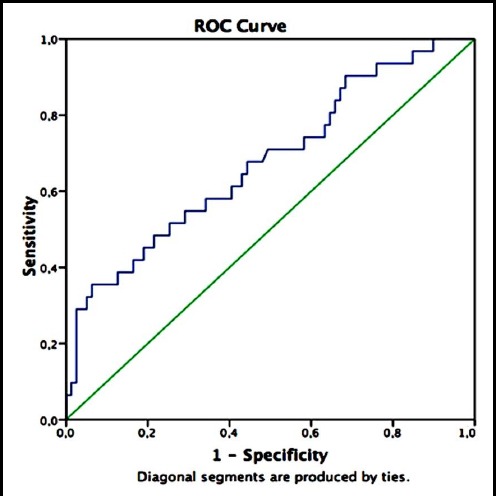


**Fig.2:**
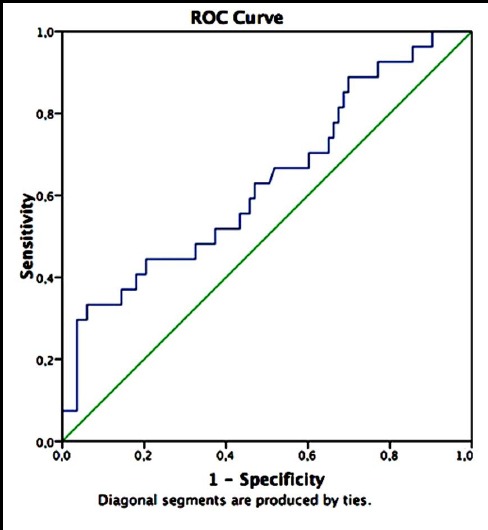


Fig.1 shows the ROC curve, which indicates the efficiency of semen presepsin values in predicting successful pregnancy. Fig.2 indicates the efficiency of semen presepsin values in predicting live birth. In the context of successful pregnancy, the area under the curve (AUC) for semen presepsin values was 0.677, 95% confidence interval (CI):[0.563-0.792];p=0.004. The most appropriate semen presepsin cut-off point in predicting successful pregnancy was 199 pg/mL. Using 199 pg/mL as the cut-off point, the sensitivity and specificity of presepsin values in predicting successful pregnancy were 64.5% and 57.0%, and their positive and negative predictive values were 37.0% and 80.4%, respectively. In the context of live birth, the AUC for semen presepsin values was 0.634, 95% CI: [0.509-0.759];p= 0.037. The most appropriate semen presepsin breakpoint in predicting live birth was again 199 pg/mL. Using 199 pg/mL as the cut-off point, the sensitivity and specificity of presepsin values in predicting live birth was 59.3% and 54.2%, and their positive and negative predictive values were 29.6% and 80.4%, respectively.

## DISCUSSION

When the studies related to presepsin are analyzed, it is clear that it represents acute inflammatory events^6^. In this study we did not measure “fresh” presepsin immediately. We think that this does not pose a problem when compared with previous studies; however, the number of similar studies is not large and perhaps this approach is not the most accurate.^11^,^12^ Although the diagnostic significance of serum presepsin measurements have been established, no reports have addressed the presence of this peptide in seminal fluid. We observed that it was possible to measure presepsin in semen and that patients can have different values. We also observed that the same patient could have different seminal plasma and plasma presepsin values. The main aim of the study was to find out a new marker that could predict successful pregnancy and/or live birth in infertile patient groups. Our ROC curve results indicate that the AUC values both in the context of successful pregnancy and live birth were 0.677 and 0.634, respectively.

As is known, in order to be a good indicator, AUC values have to be 0.80 and above. However, the small sample size in our study may have prevented us attaining such a level. As the variants in our study were not homogenous, we had to use a non-parametric test. In addition, standard deviation of semen and plasma presepsin values were found to be very high. This situation could correspond to a reconsideration of presepsin measurement standards. Nonetheless, presepsin values were found to be statistically significant in both successful pregnancy and live birth (p=0.004 and p=0.037, respectively). As a result of the ROC curve analysis, the cut-off point most appropriate for both positions was calculated as 199 pg/mL. When semen presepsin values were taken to be 199 pg/mL and above as a diagnostic value in predicting successful pregnancy and live birth, the negative predictive values were high and at a good level, 80.4%.

In conclusion, semen presepsin values could be a new diagnostic test for predicting ongoing pregnancy and/or live birth. In particular, their negative predictive value is quite strong. This prelimimary study is weak in numeric terms. Further studies with larger sample sizes are necessary to confirm our preliminary findings.
